# On Modern Progress in Ophthalmic Medicine and Surgery

**Published:** 1893-11-11

**Authors:** Robert Brudenell Carter


### On Modern Progress in Ophthalmic Medicine and Surgery.

By Robert Brudenell Carter, F.R.C.S.

The Census returns of the last twenty years display a steadily diminishing proportion of blindness in the population of England and Wales. In 1871 there were 951 blind persons to each million living, in 1881 there were 879 to each million, and in 1891 there were only 809 to each million. The improvement is distributed over all ages, but is most marked among children and elderly people; circumstances which seem to show that many of the causes of blindness operative during middle life, such as accidents or the hurtful effects of industries, are less under control than disease. Moreover, it is well known to all conversant with the subject that many states which have little tendency to produce blindness, but much tendency to render the subjects of them incapable of sustained exertion of sight, are now constantly relieved by the proper adaptation of optical appliances. I propose, in the following articles, to give some account of the steps by which these improved conditions have been reached, and to indicate what hope they afford of continued progress in the same direction. The narrative will supply incidental evidence of the nature of the methods by which alone increased power to prolong life and to diminish suffering can be attained. Experimentation guided by learning, and controlled at every stage by devotion to the interests of patients, furnishes the only means by which medical and surgical knowledge can be enlarged, or by which the difficulties of one period can be rendered contributory to the triumphs of the next.

In 1849, when I entered a medical school in London, there was not one, as far as I am aware, in which any useful instruction was given with regard to affections of the eye. At the London Hospital, where I was a pupil, I never saw a case of eye disease either in the wards or among the out-patients; and the only information afforded me on the subject was comprised in six lectures which formed part of the general course on surgery, and which were illustrated, in the absence of patients, by a few coarsely executed drawings in water colour. The idea that a question on the eye might be asked in examination at the College of Surgeons was one which never presented itself even to the imagination of a student. Sir Thomas Watson, in his Lectures on the Principles and Practice of Physic, had, indeed, dealt in his usual masterly way with the forms of eye disease which fell within his province; and had used the visible course of iritis and of conjunctivitis to explain the analogous, but hidden, changes which occurred in bronchitis or in pleurisy. As far as practice was concerned, the public work was entirely done in "special" hospitals; of which only the Royal London Ophthalmic, founded in 1804, had obtained any considerable reputation. The Royal Westminster was of almost equal antiquity, having been founded in 1816 by Mr. Guthrie, soon after his retirement from the army; but it long retained the character rather of a private than of a public institution. The Central London was still young, having been founded in 1843; and these were all. It was impossible for medical students, generally speaking, to attend the practice of the special hospitals, on account of the time which such attendance would consume; and hence they were admitted to qualify, and went into practice, without even such knowledge of eye disease as then existed. My own ignorance was brought home to me in a very direct and unpleasant manner. I was serving in the Crimea, during the Russian war, when one of my eyes became severely inflamed. I neither knew what was the matter with it nor how it should be treated; and the acute perceptions of a sufferer showed me that the medical colleagues who were at hand, however liberal they might be in the use of phrases, were in reality no better informed than myself. The period of anxiety through which I passed led me to form the resolution that, if I returned safely to England, I would endeavour to render myself fit for professional responsibilities in so far as the organs of vision were concerned.

A few years previously, about 1848, the late Mr. Charles Babbage had discovered that, by means of a mirror with a central perforation, it was possible to see every detail of the interior of the eye. His instrument was the "ophthalmoscope," which was destined to bring about a revolution in ophthalmic surgery. But a prominent London specialist assured him that the invention would be useless, and Babbage threw it aside. Four years later, Helmholtz repeated Babbage's discovery with a contrivance inferior in itself, but which fell into the appreciative hands of Von Graefe. Its illuminating power was found to be insufficient, and then Ruete reinvented the precise instrument of Babbage. From this course of events it followed, not unnaturally, that German practitioners enjoyed a priority in the use of the ophthalmoscope, and that they arrived at many discoveries by its aid before its value was fully realised in other countries. The advantages thus gained could only be temporary; and a stirring of the English medical mind, in relation to ophthalmology, soon began to make itself apparent The late Mr. Zachariah Laurence, who possessed the advantage, then somewhat unusual in the surgical profession, of being able to deal with the optical problems of vision from a mathematical standpoint, returned to London from the Continental schools in 1856, and endeavoured to obtain office in one of the special hospitals already mentioned. But these institutions were very close boroughs, in which mere knowledge was held to be a somewhat secondary consideration; and hence Mr. Laurence, in 1857, found it necessary to his purposes to establish the South London Ophthalmic Hospital, where he worked strenuously at the development of the branch of surgery which he had chosen to pursue. To some of his results it will be necessary to refer hereafter; but, in the meanwhile, it may be observed that, as so often happens, the darkest period was that just before the dawn. Of this, perhaps, a single illustration may suffice. The senior surgeon to the Royal London Ophthalmic Hospital published a "Practical Guide," or some such title, to the diseases of the eye, with a chapter on the ophthalmoscope. He was so absolutely ignorant of the elements of optics, and of the mode of formation of images by lenses, that he did not know whether the picture shown by the ophthalmoscope was erect or inverted, and discussed the question as one in dispute, and which might be settled by guessing, without having the faintest notion that erectness or inversion depended upon the manner in which the instrument was used.

